# Spectroscopic Active Probes for Investigation of Lipid Transformation in Cells and Membranes

**DOI:** 10.1021/acs.jpcb.5c05981

**Published:** 2026-01-27

**Authors:** K. Chrabąszcz

**Affiliations:** Institute of Nuclear Physics, Polish Academy of Sciences, Radzikowskiego 152, 31-342 Krakow, Poland

## Abstract

Deuterium-labeled lipids provide a powerful means to probe lipid organization, dynamics, and molecular interactions in complex biological systems. In this work, systematic spectroscopic characterization of deuterated fatty acids, sterols, phospholipids, and sphingolipids using Raman and infrared (IR) spectroscopies is presented. Importantly, this study establishes a fundamental spectroscopic reference framework and analytical guidelines for investigating lipid transformation processes in cells and membranes, as all data presented herein are acquired from well-defined lipid standard compounds. The unique C–D stretching region (2300–2000 cm^–1^), located within the spectroscopically “silent” window and absent in endogenous cellular components, was exploited for selective detection and semiquantitative assessment of lipid probes. Integration of C–D band intensities enables the estimation of the probe content in complex matrices as the signal correlates with the degree of deuterium substitution. While the C–D spectral region remained largely invariant with respect to the lipid backbone structure, other vibrational modes, particularly those associated with headgroups or specific functional moieties, exhibited lipid-class-dependent intensity variations upon deuteration, reflecting differences in the molecular environment and vibrational coupling. Importantly, deuteration does not significantly interfere with biological function, as replacing hydrogen with deuterium only marginally increases the atomic mass without altering the chemical structure, polarity, or overall molecular interactions, thereby preserving the native lipid behavior. The reference spectra presented here provide an essential foundation for interpreting hyperspectral vibrational data acquired from cellular and membrane systems, supporting future applications of label-free Raman and IR imaging aimed at monitoring lipid remodeling, metabolic regulation, and therapeutic modulation in biological contexts.

## Graphical abstract

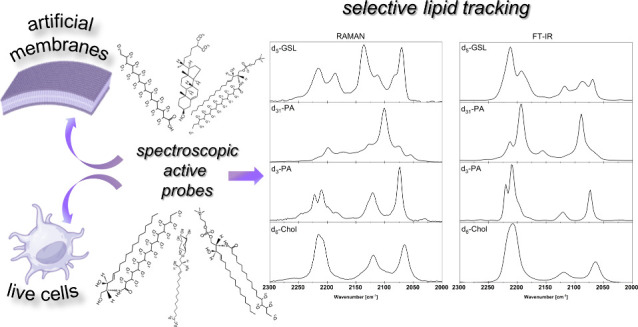

## Introduction

Cellular biochemical pathways orchestrate essential physiological and pathological processes through the coordinated actions of numerous interacting and regulatory molecules.^1–3^ Among these, lipids play a central role not only in maintaining the membrane architecture and energy balance but also in modulating signaling networks and stress responses. Altered lipid metabolism is now recognized as a hallmark of multiple diseases, including cancer, neurodegeneration, cardiovascular disorders, diabetes, and inflammation.^4–8^ Such dysregulation influences membrane fluidity, organelle communication, and cellular adaptation, often contributing to disease progression and therapy resistance.^9–11^

In cancer biology, lipid reprogramming has been closely linked to radioresistance, where changes in lipid droplet (LD) abundance and membrane composition affect cellular responses to ionizing radiation.^12,13^ LD accumulation also correlates with altered iron metabolism, implicating oxidative stress and DNA repair capacity in radioresistant cells.^14^ These findings highlight the need to investigate lipid remodeling not only within intracellular reservoirs but also within the plasma membrane, the critical interface for environmental interactions and stress signaling.

A variety of analytical methods, including mass spectrometry (MS), nuclear magnetic resonance (NMR), and fluorescence-based imaging, have been developed to study lipid metabolism.^15–18^ While each provides valuable molecular or spatial information, these approaches often require extensive sample preparation, labeling, or complex data analysis. Consequently, there is a growing interest in vibrational spectroscopy as a label-free, nondestructive alternative capable of directly probing lipid chemical structures and their transformations.

Recent advances in Raman spectroscopy (RS) and Fourier-transform infrared (FT-IR) spectroscopy combined with deuterium labeling have opened new opportunities for highly specific lipid tracking. Deuterated molecules exhibit distinct C–D stretching vibrations in the “silent” spectral window (1800–2800 cm^–1^), where endogenous biomolecules contribute minimal background.^19–22^ This feature enables the selective identification and monitoring of specific lipid subclasses within complex biological environments, overcoming the key limitations of conventional labeling methods. It has been unequivocally demonstrated that deuterated fatty acids enable precise tracking of lipid metabolism and transformation products in living cells by Raman spectroscopy.^23,23^ Using the characteristic C–D stretching vibrations located in the silent Raman window (∼2100–2300 cm^–1^), deuterium-labeled fatty acids can be selectively visualized against endogenous lipid pools, allowing the quantification of their incorporation into lipid droplets and monitoring of downstream metabolic conversions such as desaturation and esterification through measurable shifts in the C–D spectral profile. Redistribution of the C–D signal across membrane compartments and lipid droplets further makes it possible to capture the formation of metabolites including phospholipids and cholesteryl esters, while β-oxidation manifests as a progressive decrease in C–D band intensities. Evidence from complementary vibrational spectroscopy studies confirms the robustness of this approach, showing that excess exogenous fatty acids, deuterated or not, accumulate in lipid droplets and that deuterated arachidonic acid enables clear spectral distinction between endogenous cellular lipids (∼1661 cm^–1^) and exogenous pools (∼1637 cm^–1^),^24^ while IR spectroscopy of endothelial and leukemic cells treated with d_31_-palmitic acid detects characteristic C–D bands fully consistent with reference spectra despite intracellular lipid rearrangement.^25^ Raman hyperspectral imaging has also been applied to investigate lipid-dependent cytotoxicity in cancer, where tracking of all-deuterated γ-linolenic acid demonstrated that tumor-selective toxicity originates from preferential lipid droplet accumulation and can be monitored using both C–D and deuterated C=C vibrational markers.^26^ More recent methodological developments have introduced computational spectral unmixing to extract individual deuterated fatty acids from their intracellular mixtures^27^ and demonstrated that the gauche/trans conformational ratio of deuterated phospholipids directly modulates Raman bands in a viscosity-dependent manner, enabling the quantitative estimation of intracellular lipid biophysical states.^21^

In this context, this work establishes a spectroscopic reference framework and analytical guidelines for investigating lipid organization and transformation in cellular and membrane systems. A comprehensive Raman and IR spectral library of major lipid classes such as fatty acids, cholesterol and its esters, phospholipids, and sphingolipids, together with their deuterated analogues, is presented. Systematic analysis of their vibrational fingerprints, with particular emphasis on the C–D stretching region (∼2300–2000 cm^–1^), provides reliable benchmarks for lipid identification and semiquantitative evaluation. These spectra serve as a foundation for interpreting hyperspectral vibrational data acquired from complex biological environments. Consequently, the data set supports future applications of label-free vibrational imaging to investigate lipid remodeling associated with pathological processes, cancer progression, and therapeutic response across artificial, cellular, and subcellular length scales.

## Experimental Section

### Materials and Methods

#### Spectroscopic Probes

Selected lipids with their deuterated forms were purchased from Sigma-Merc/Avanti Research according to Table 1.

**Table 1:** List of Selected Lipids and Their Deuterated Forms with Abbreviations and Product Numbers

no.	compound name	abbreviation	product number	physical state (room temp.)
***Fatty acids***				
1.	Palmitic acid	PA	P0500	solid
2.	Palmitic acid d_31_	PA-*d*_31_	366897	solid
3.	Palmitic acid d_3_	PA-*d*_3_	315951	solid
4.	Stearic acid	SA	85679	solid
5.	Stearic acid d_35_	SA-*d*_35_	448246	solid
6.	Oleic acid	OA	O1008	liquid (oil)
7.	Oleic acid d_9_	OA-*d*_9_	861809O	liquid (oil)
8.	Linoleic acid	LA	L1376	liquid (oil)
9.	Linoleic acid d_5_	LA-*d*_5_	700364O	liquid (oil)
10.	Arachidonic acid	AA	861810E	liquid (oil)
11.	Arachidonic acid d_11_	AA-*d*_11_	861809O	liquid (oil)
***Cholesterol and esters***				
12.	Cholesterol	Chol	C3045	solid
13.	Cholesterol d_6_	Chol-d_6_	700172P	solid
14.	Cholesteryl palmitate	Chol-PA	C6072	solid
15.	Cholesteryl palmitate d_7_	Chol-PA-*d*_7_	700149P	solid
***Phospholipids***				
16.	Phosphatidylethanolamine	PE	P7943	solid
17.	Phosphatidylethanolamine d_31_	PE-*d*_31_	86037	solid
18.	Phosphatidylcholine	PC	P1138	solid
19.	Phosphatidylcholine d_70_	PC-*d*_70_	860365P	solid
20.	Phosphatidylcholine d_83_	PC-*d*_83_	860368P	solid
21.	Phosphatidylserine	PS	840032P	solid
22.	Phosphatidylserine d_31_	PS-*d*_31_	860403P	solid
***Sphingolipids***				
23.	Sphingomyelin	SM	85615	solid
24.	Sphingomyelin d9 (18:0)	SM-*d*_9_	791649	solid
25.	Sphingomyelin d31 (16:0)	SM-*d*_31_	868584P	solid
26.	Ceramide	CER	43799	solid
27.	Ceramide d31	CER-*d*_31_	868516P	solid
28.	Glycosphingolipid	GSL	860441P	solid
29.	Glycosphingolipid d5	GSL-*d*_5_	860636P	solid

Oil samples were directly deposited on CaF_2_ windows for spectroscopic measurements. All powdered compounds were dissolved in ethanol (cholesteryl palmitate in THF) to prepare saturated solutions; then, 20 μL of the solution was deposited on CaF_2_ and subsequently purged with a gentle N_2_ flux before measurements to ensure complete solvent evaporation and a good spectra S/N ratio.

#### Raman Measurements

The Raman spectra of all compounds from Table 1 were recorded using a Renishaw InVia Raman spectrometer equipped with an optical confocal microscope, an air-cooled solid-state laser emitting at 532 nm (10% laser power), 633 nm (100% laser power), and 785 nm (100% laser power), and a CCD detector cooled to −70 °C. During measurements, no sample damage was observed. An air (20×, 0.45, Nikon) objective was used. Lipids and their deuterated forms were deposited on CaF_2_ and spectra were recorded for each laser line in five points, with 10 s of integration time, 1 accumulation, and a spectral resolution of ∼2 cm^–1^.

#### FT-IR Measurements

Infrared spectra collection of all compounds from Table 1 was performed using a Thermo Scientific Nicolet iN10 MX microscope operating in transmission mode. The system featured a permanently aligned 15× objective and a liquid nitrogen-cooled MCT-A linear array detector, enabling spectral acquisition within the 3800–800 cm^–1^ range. Spectra were acquired at a resolution of 4 cm^–1^, with 256 scans accumulated per measurement. The air background (256 scans accumulated) was recorded before each sample spectrum collection.

#### Data Treatment and Visualization

Raman and IR data were analyzed using WiRE (ver. 5.3, Renishaw, United Kingdom) and OPUS (ver. 7.5, Bruker Optics, Germany) software, respectively. The mean spectra of each compound were obtained by averaging single spectra collected from 5 points after performing baseline correction and normalization. Integral intensities of C–D bands were calculated in the range of ∼2200–2000 cm^–1^, depending on the investigated compound, in OPUS software. OriginPro (ver. 2024) software was used for data visualization. To enhance the visibility of specific features, some spectra were offset and magnified in selected wavenumber regions. The correlation of Raman spectra of all investigated compounds, recorded with 633 and 785 nm, can be found in the Supporting Information (Figures S1–S4).

## Results and Discussion

### Fatty Acids: Structure and Cellular Function

Fatty acids are long-chain hydrocarbons with a terminal carboxylic acid group and represent fundamental building blocks of complex lipids.^28^ Their structural diversity, defined by the chain length, degree of unsaturation, and configuration, influences the physicochemical properties of cellular metabolism, membranes, and lipid-derived signaling. Saturated fatty acids typically contribute to membrane rigidity, whereas unsaturated fatty acids introduce turns into the hydrocarbon chains, increasing membrane fluidity. Beyond their structural roles, free fatty acids and their oxidized derivatives function as signaling molecules modulating inflammation, oxidative stress, apoptosis, and mitochondrial function.^29,30^ In cancer, dysregulation of fatty acid metabolism supports rapid proliferation and survival under metabolic stress.^31^ Alterations in fatty acid composition have also been linked to therapy resistance, highlighting their importance as both functional membrane constituents and bioactive mediators.^32^

The spectral patterns of representative fatty acids, including the saturated palmitic acid (PA) and stearic acid (SA) and the unsaturated oleic acid (OA), linoleic acid (LA), and arachidonic acid (AA), were correlated (Figure 1). The spectra of these compounds overlap in both regions, 3000–2000 cm^–1^ and 1800–600 cm^–1^, and thus coincide with those of complex biological samples or other constituents used in liposome or membrane preparation. To enable selective spectroscopic tracking of these lipid fractions, we examined their deuterated analogues.

**Figure 1:**
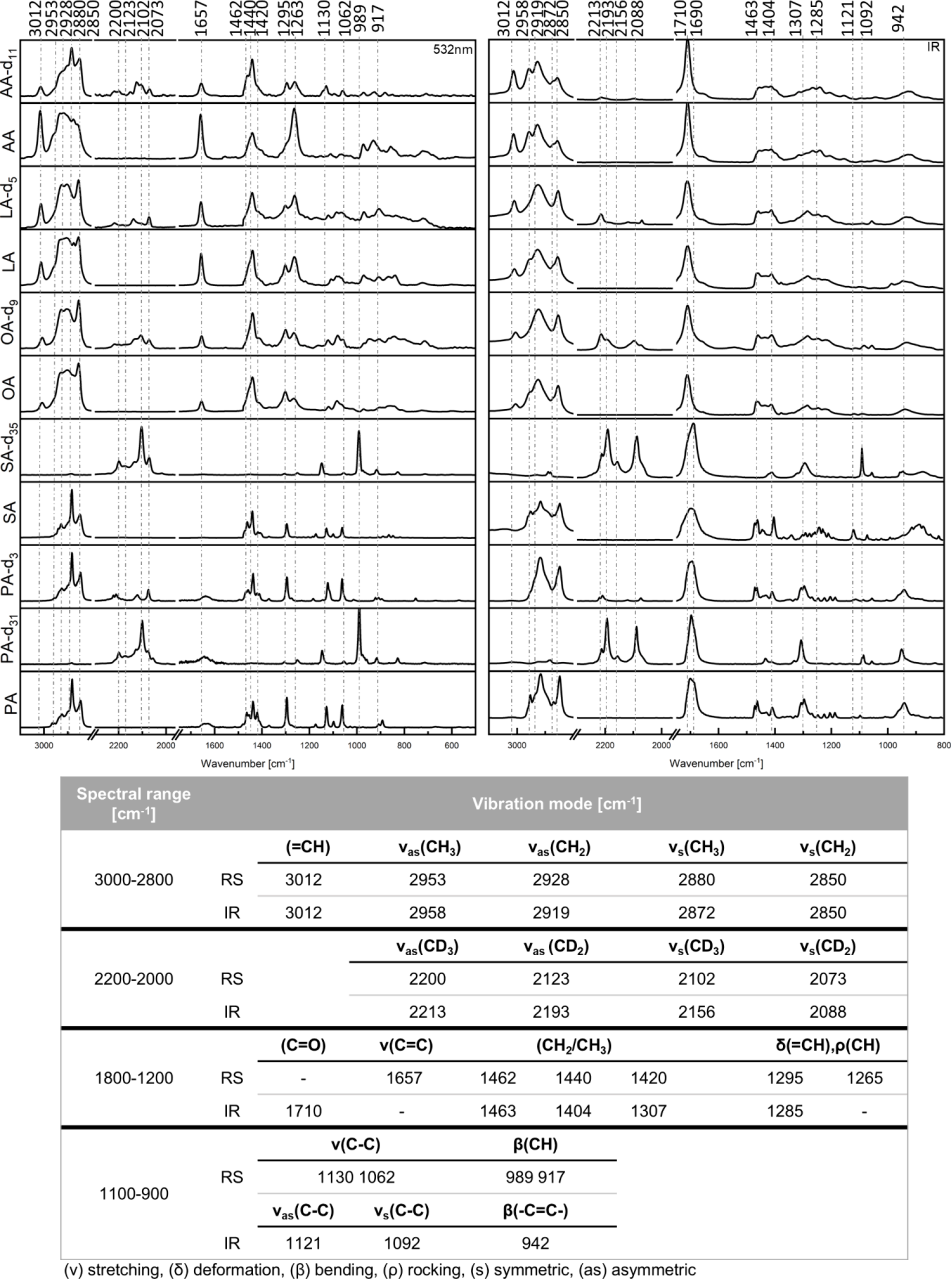
Representative vibrational spectra of selected fatty acids and their deuterated forms acquired using Raman (left, 532 nm laser line) and FT-IR (right) spectroscopies, with key band assignments highlighting characteristic molecular vibrations.^33,21,34,35^

For PA and SA, the Raman and IR profiles are highly similar, because they differ primarily in the acyl chain length (C_16_ vs C_18_). Complete substitution of all ^1^H atoms with ^2^H shifts the C–H stretching vibrations from the 3000–2800 cm^–1^ to the 2200–2000 cm^–1^ region, as observed for PA-*d*_31_ and SA-*d*_35_. Such a substitution also influences −CH_2_ (1462 cm^–1^) and −CH_3_ (1440 cm^–1^) deformation bands, which are no longer observed in the Raman spectra. A band at 989 cm^–1^ appears, reflecting the expected isotopic downshift of −CH_2_ vibrations upon −CD_2_ substitution. In deuterated PA and SA, the −C=O band shifts from 1710 to 1690 cm^–1^, and the C–C stretching at 1092 cm^–1^ becomes more intense. Partial deuteration (PA-*d*_3_) caused no major spectral changes, although weak C–D signals remained visible at 2200–2000 cm^–1^ in both techniques.

For unsaturated fatty acids, the most distinctive Raman feature was the =CH stretching band at ∼3012 cm^–1^, whose intensity increases with the number of double bonds (OA < LA < AA) also for C=C stretching band at 1657 cm^–1^. Fully deuterated derivatives (PA-*d*_31_, SA-*d*_35_) display distinct C–D stretching patterns compared to partially substituted OA-d_9_, LA-d_5_, and AA-d_11_. Differences appear in both the band presence and relative intensity, especially within 2100–2050 cm^–1^ (e.g., 2121 vs 2105 cm^–1^ for OA-d_9_ and AA-d_11_; Figure S5A). In the IR spectra of unsaturated FAs, the =CH band at ∼3012 cm^–1^ is visible, although variations in the C–D region are less pronounced than in the Raman spectra. (Figure S5A, IR spectra).

The clear dependence between the deuterium substitution level and the C–D band intensity demonstrates that deuterated fatty acids can serve as sensitive spectroscopic markers of lipid remodeling and oxidation processes. These spectral characteristics contribute to the reference library of deuterated lipid probes, enabling the identification of fatty acid subclasses and their transformations in both model membranes and living cells through label-free Raman and infrared imaging.

### Cholesterol and Esters: Membrane Regulators and Lipid Storage

Cholesterol is a key sterol lipid abundant in eukaryotic plasma membranes, where it modulates membrane order, stiffness, and permeability.^36,37^ Its planar, rigid ring structure intercalates between phospholipid fatty acid tails, promoting tighter packing and stabilizing lipid raft domains, cholesterol-enriched microdomains that facilitate membrane protein organization and signal transduction.^38,39^ Cells tightly regulate cholesterol levels via synthesis, uptake, and esterification.^40^ Cholesteryl esters, the storage form of cholesterol, are sequestered in intracellular lipid droplets and mobilized under metabolic demand.^41^ In cancer, elevated levels of cholesteryl esters are associated with enhanced membrane biogenesis, lipid signaling, and protection from oxidative stress.^42^ Their accumulation is often linked to tumor aggressiveness, metastasis, and resistance to radiotherapy or chemotherapy.^43^ Thus, understanding cholesterol trafficking and esterification dynamics is crucial for elucidating membrane remodeling and adaptive survival mechanisms in cancer cells.

For spectroscopic analysis, spectral profiles of cholesterol and cholesteryl palmitate with their deuterated analogues were investigated (Figure 2). In both cases, deuterium substitution was introduced into the end of the sterol ring side chain, incorporating six atoms in cholesterol d_6_ (Chol-d_6_) and seven in cholesteryl palmitate-d_7_ (Chol-PA-d_7_).

**Figure 2:**
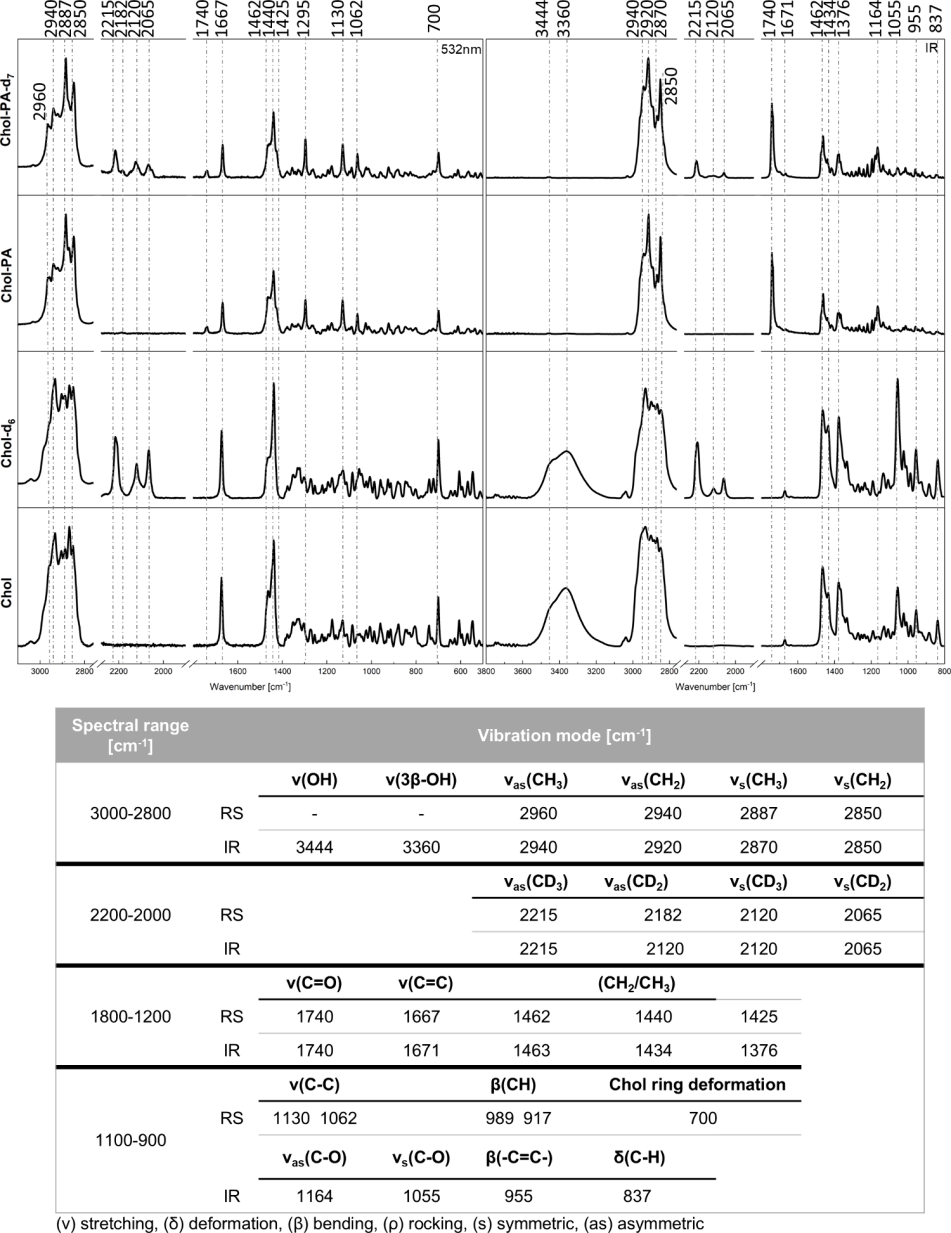
Representative vibrational spectra of cholesterol and cholesteryl palmitate with their deuterated forms acquired using Raman (left, 532 nm laser line) and FT-IR (right) spectroscopies, with key band assignments highlighting characteristic molecular vibrations.^33,21,34,35,44^

The Raman spectrum of native cholesterol exhibited characteristic features at 2940 and ∼2864 cm^–1^, arising from −CH_2_ and −CH_3_ symmetric stretching at the chain terminus, respectively. A band at 1667 cm^–1^ corresponds to −C=C stretching within the sterol ring, while the 700 cm^–1^ signal is attributed to in-plane −C=C deformation of the ring. Isotopically labeled Chol displayed an analogous pattern, with the addition of distinct C–D stretching bands at 2215, 2120, and 2060 cm^–1^ (Figure S5B). Notably, unlike certain fatty acids (Figure 1), deuteration did not induce any significant band enhancement in the Raman spectrum.

In the IR spectrum, cholesterol shows intense, broad absorptions at 3444 and 3360 cm^–1^, assigned to the −OH group. This single hydroxyl moiety is attached to the steroid ring structure and exhibits polar and hydrophilic properties. Another prominent IR feature was the 1055 cm^–1^ band from C–O stretching vibrations. Deuterium incorporation produces additional absorptions in the 2200–2000 cm^–1^ range (2213, 2180, and 2065 cm^–1^), consistent with C–D stretching, and significantly enhanced the intensity of the 1055 cm^–1^ band.

Upon esterification to cholesteryl palmitate, a −C=O ester band appeared at 1740 cm^–1^ in both techniques; however, it was stronger in IR due to the large dipole moment change of the polar −C=O bond during vibration, while its weak polarizability change yields a comparatively weak Raman signal. Concurrently, IR spectra showed the disappearance of the −OH bands, reflecting the loss of the hydroxyl group, along with intensified −CH_3_ and −CH_2_ vibrations arising from the palmitic acid chain. In the Raman spectrum of Chol-PA-d7, in addition to the main C–D bands at 2215, 2120, and 2050 cm^–1^, weak shoulders appeared at 2182, 2150, and 2050 cm^–1^. In the IR spectrum, the 2200–2000 cm^–1^ region of Chol-PA-d7 closely resembled that of Chol-d_6_, with the exception of a weak shoulder at 2180 cm^–1^ (Figure S5B).

The distinct vibrational profiles of cholesterol and its ester highlight the sensitivity of C–D and C–H bands to the molecular environment and packing order. This information enriches the spectral database of lipid fingerprints, supporting the precise detection of cholesterol redistribution and esterification in biological membranes, both in vitro and in living systems, through noninvasive spectroscopic methods.

### Phospholipids: Structural Scaffolds and Signaling Mediators

Phospholipids are amphipathic molecules forming the bilayer architecture of cellular membranes, where variations in headgroups and acyl chains enable structural specialization, lateral heterogeneity, and dynamic remodeling.^45,46^ Disruption of their composition or metabolism is common in malignancies, contributing to altered signaling, vesicle transport, and therapy resistance.^47–49^

Phosphatidylethanolamine (PE), phosphatidylcholine (PC), and phosphatidylserine (PS) are major classes of glycerophospholipids that differ in their polar head groups, influencing membrane structure and function.^50^ While PC and PE are zwitterionic at physiological pH, PS carries a net negative charge due to the carboxyl group in serine, contributing to the membrane surface potential and signaling roles. Moreover, PE and PS are predominantly localized in the inner leaflet of the plasma membrane, whereas PC is typically enriched in the outer leaflet, reflecting their distinct roles in membrane asymmetry and cellular processes such as apoptosis and vesicle trafficking.^49^ PC, PE, and PS share a common glycerol backbone with two fatty acid chains.^50^ The hydrophobic fatty acid chains determine membrane fluidity and length, while the distinct polar headgroups (choline, ethanolamine, or serine) influence the membrane charge, curvature, and interactions.

The collected Raman and IR spectra of PC, PE, and PS enabled a clear differentiation of these phospholipids based on their distinct spectral profiles (Figure 3). Differences in C–H stretching vibrations, arising from variations in the fatty acid chain composition, strongly influence the band shapes in the 3000–2800 cm^–1^ region, where contributions from −CH_2_ and −CH_3_ groups dominate. Notably, PE and PS exhibited a band at 3013 cm^–1^, corresponding to =CH stretching vibrations in unsaturated fatty acid chains, accompanied by the 1657 cm^–1^ band assigned to −C=C stretching modes, similar to IR. This spectral pattern was absent in PC, consistent with the lack of double bonds in the examined compound. In the high-wavenumber region of the IR spectra, PC showed a broad band at approximately 3382 cm^–1^, attributed to −OH stretching vibrations, most likely from trace amounts of tightly bound water associated with the polar headgroup. As all three discussed phospholipids contain phosphate groups, in each IR spectrum, bands related to asymmetric (∼1240 cm^–1^) and symmetric (∼1080 cm^–1^) vibrations occur. A characteristic feature of PC is the presence of a sharp 1470 cm^–1^ band from the methyl group attached to the nitrogen of the choline moiety. The IR band at ∼970 cm^–1^, assigned to the −P–O–C stretching between the phosphate group and the choline/serine moiety, is characteristic for PC and PS, respectively. In contrast, PE displays a broad band at ∼1074 cm^–1^ arising from symmetric C–O vibration and a small shoulder at ∼1030 cm^–1^ from C–N vibration, from the ethanolamine moiety.

**Figure 3:**
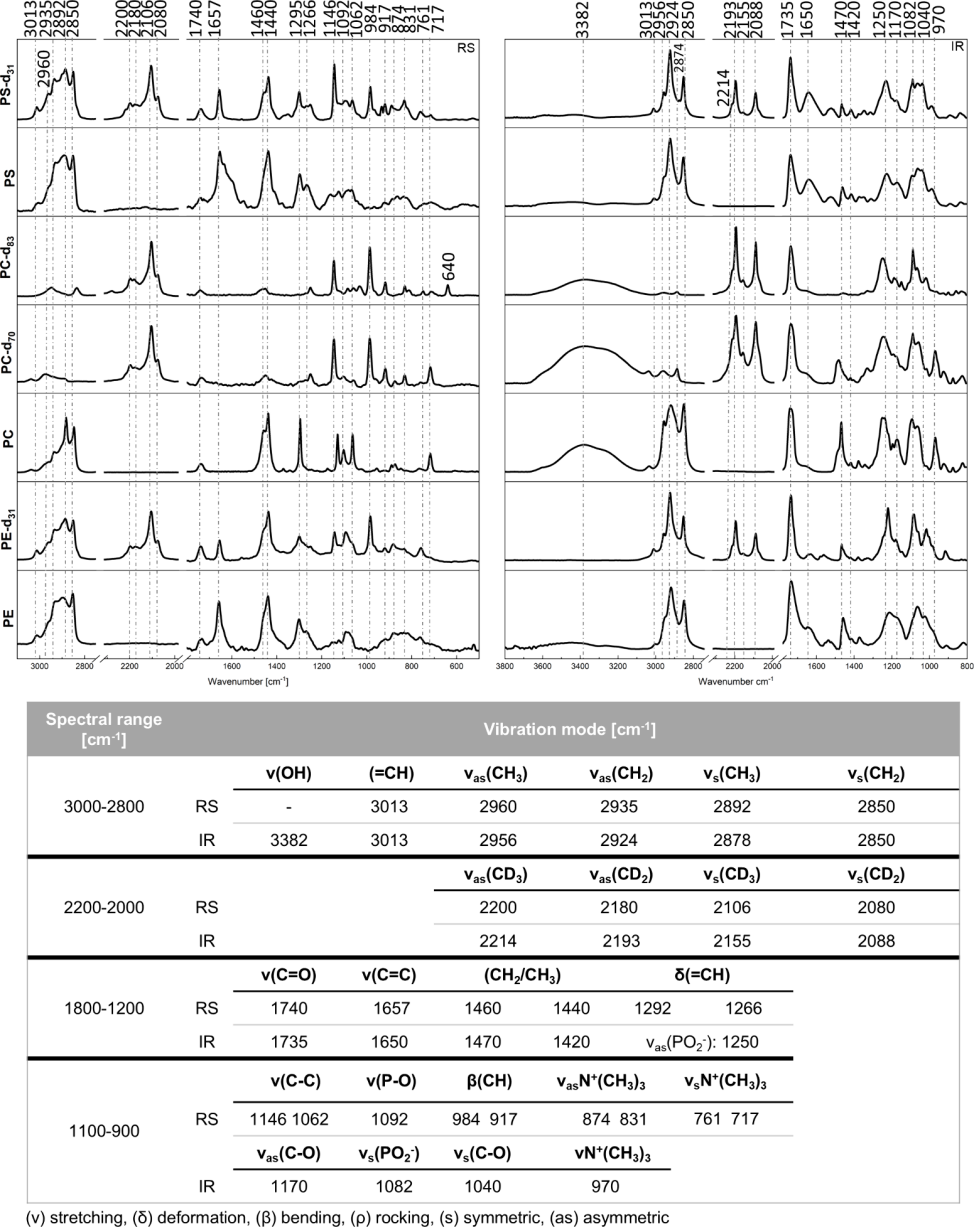
Representative vibrational spectra of selected phospholipids with their deuterated forms acquired using Raman (left, 532 nm laser line) and FT-IR (right) spectroscopies, with key band assignments highlighting characteristic molecular vibrations.^33,44,51–53^

Three isotopic substitution schemes were investigated. In PE, one fatty acid chain was partially deuterated, replacing 31 hydrogen atoms with deuterium, while the second chain remained unchanged. For PC, two labeling patterns were examined: in PC-*d*_70_, all hydrogens in the fatty acid chains were substituted; in PC-*d*_83_, the fatty acid chains were fully deuterated, and additional hydrogens in the choline headgroup were also replaced. For PS, the labeling scheme matched that of PE, with 31 deuterium atoms incorporated into one fatty acid chain.

In PE, single-chain deuteration resulted in C–D stretching bands between 2300 and 2000 cm^–1^, displaying a profile similar to that of deuterated fatty acids (Figure 1). Isotopic substitution enhanced the intensity of the 1657 cm^–1^ band and introduced additional features at 1146 and 984 cm^–1^, attributed to −CD_2_ twisting modes, also observed in fully deuterated palmitic (d_31_) and stearic (d_35_) acids (Figure 1). The IR spectrum of PE-*d*_31_ exhibited a similar pattern, with an additional band at ∼1250 cm^–1^ from shifted −CD_2_ vibrations.

In the Raman spectra of PC-*d*_70_, substitution of both fatty acid chains caused a red shift of −CH_3_ and −CH_2_ stretching modes from 3000–2800 to 2200–2000 cm^–1^, accompanied by the disappearance of bending, scissoring, and twisting modes at 1460, 1440, and 1295 cm^–1^, respectively. Moreover, C–C stretching bands at 1164 and 1062 cm^–1^ were also red-shifted. Interestingly, in PC-*d*_83_, where the choline headgroup was additionally deuterated, a distinct band at 640 cm^–1^ emerged, dominated by phosphate group vibrations. A comparative analysis of the IR spectra of PC-*d*_70_ and PC-*d*_83_ revealed differences in the 2200–2000 cm^–1^ region and in other spectral markers: PC-*d*_70_ showed a reduction in the 1470 cm^–1^ band, whereas in PC-*d*_83_, this band was absent, along with the disappearance of the ∼970 cm^–1^ feature. The loss of this IR band in fully deuterated PC confirms its assignment to −CH_2_ wagging vibrations, which vanish upon complete hydrogen-to-deuterium substitution.

In PS-*d*_31_, Raman spectra displayed isotope-induced effects similar to those of PE-*d*_31_, with a decrease in the 1657 cm^–1^ band intensity and an increase in the 1146 and 984 cm^–1^ features. In the IR spectra, the most prominent change was the appearance of well-defined C–D stretching bands in the 2200–2000 cm^–1^ region (Figure S5C).

The C–D band intensities in phospholipids confirm their suitability as semiquantitative markers of membrane composition and dynamics. These findings expand the spectroscopic reference framework, facilitating the discrimination of phospholipid subclasses and enabling high-resolution mapping of their distribution and remodeling in cellular and subcellular membranes using Raman and IR imaging techniques.

### Sphingolipids: Structural Integrity and Stress Signaling

Sphingolipids constitute a diverse family of lipids characterized by a sphingoid base backbone, commonly sphingosine, instead of the glycerol found in phospholipids.^50^ These lipids play critical roles in maintaining the membrane architecture and contribute to signal transduction by influencing the organization and mobility of membrane receptors.^54,55^ Major representatives include ceramides (CER), sphingomyelin (SM), and glycosphingolipids (GSL), each playing distinct structural and functional roles. Ceramides function as signaling molecules that regulate cell differentiation, proliferation, apoptosis, and responses to stress.^56,57^ Sphingomyelin contributes to the membrane structure and modulates signaling by influencing lipid raft organization and receptor clustering.^58,59^ Glycosphingolipids mediate cell–cell recognition, adhesion, and signal regulation, playing important roles in immune and inflammatory pathways.^60,61^

SM, CER, and GSL share a common sphingoid base linked to a fatty acid via an amide bond, forming a ceramide core. They differ in their head groups: sphingomyelin contains a phosphocholine moiety, glycosphingolipids carry one or more sugar residues, while ceramides lack additional polar head groups beyond the hydroxyl group on the sphingoid base.

The Raman spectrum of sphingomyelin (SM) exhibited characteristic bands of −CH_3_ and −CH_2_ vibrations (2970–2850, 1460, 1440, 1095, and 1063 cm^–1^), predominantly arising from the fatty acid chains, along with a C=C stretching band at 1672 cm^–1^ (Figure 4). The band observed at ∼718 cm^–1^ in the Raman spectrum of sphingomyelin originates from the symmetric stretching vibration of the N^+^(CH_3_)_3_–C group in the phosphocholine head, serving as a characteristic marker of choline-containing lipids. In the IR spectrum, the amide I (1645 cm^–1^, −C=O stretching) and amide II (1542 cm^–1^, −CN stretching) bands are observed, together with an −NH stretching band at 3289 cm^–1^, all attributed to the SM amide group. For the earlier described phospholipids, the influence of the deuterium substitution site on the spectral profile was examined, and in SM, the effect of the deuterium quantity was assessed. Substitution of nine deuterium atoms at the terminal end of one fatty acid chain led to the appearance of weak −CD_3_ and −CD_2_ bands in the 2200–2000 cm^–1^ region in both Raman and IR spectra. In SM-*d*_31_, where one fatty acid chain is fully deuterated, these −CD_3_ and −CD_2_ bands show marked enhancements in both techniques. Additional increases in band intensity were also evident at 1145, 983, and 718 cm^–1^ (Raman) and 1080 and 970 cm^–1^ (IR), corresponding to phosphocholine vibrations.

**Figure 4:**
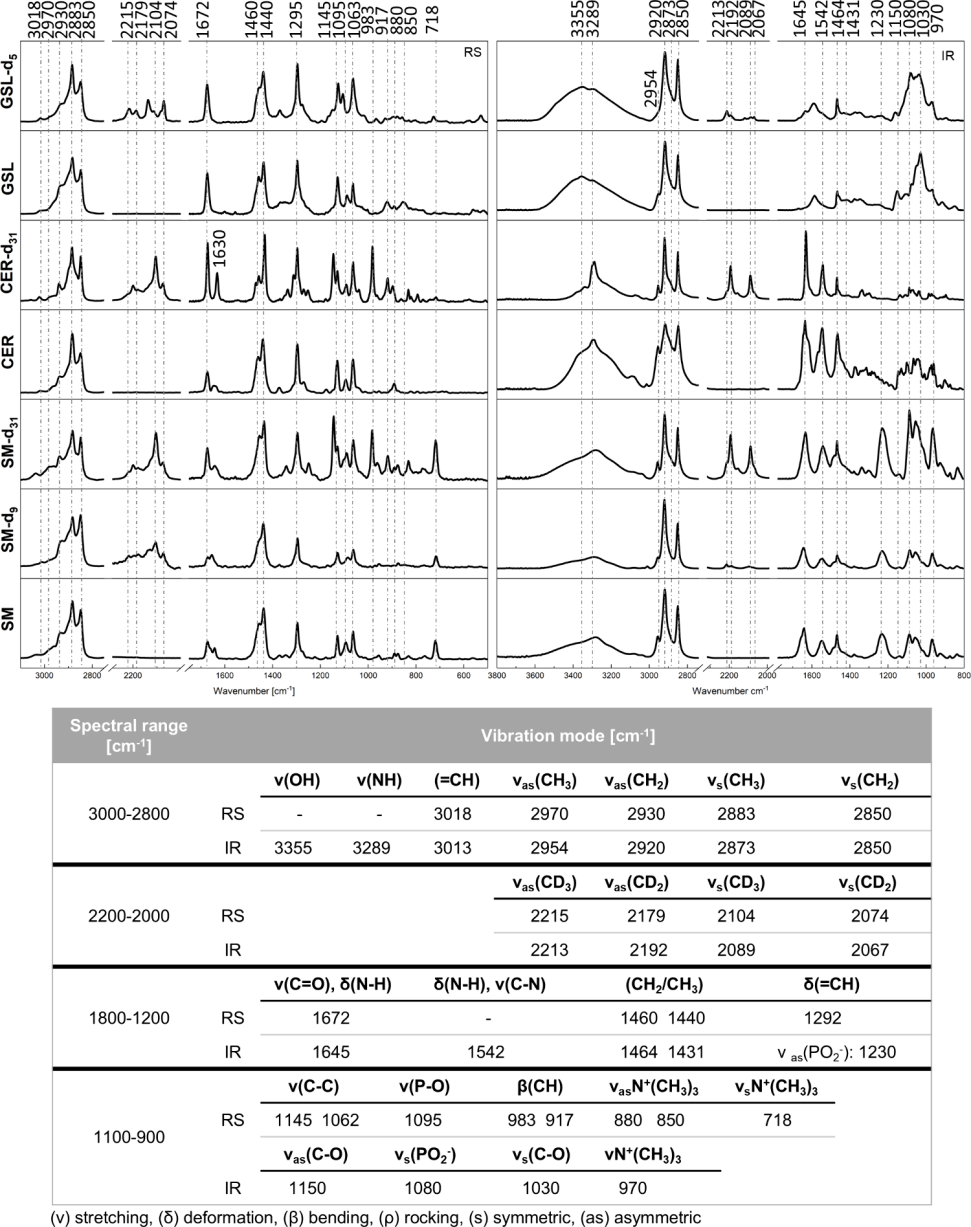
Representative vibrational spectra of selected sphingolipids with their deuterated forms acquired using Raman (left, 532 nm laser line) and FT-IR (right) spectroscopies, with key band assignments highlighting characteristic molecular vibrations.^33,51,44,62^

The Raman and IR spectra of CER displayed a band composition similar to that of SM due to their analogous molecular backbones but lack signals from phosphate and choline groups. When 31 deuterium atoms were incorporated, the Raman spectrum closely resembled that of SM-*d*_31_, displaying intense C–D bands in both the 2200–2000 and 1145–917 cm^–1^ regions. The corresponding IR spectrum showed a similar pattern except for the absence of the 1080 and 970 cm^–1^ bands. In CER, a prominent −NH stretching band (amide A) at 3289 cm^–1^ was observed with increased intensity after deuteration.

The Raman spectrum of GSL contains the same fatty acid chain and −C=C vibration bands as SM and CER. However, the IR profile differs due to the carbohydrate (glycosyl) moieties in the GSL structure. The −C–O–C stretching vibrations in sugars are strong in IR spectra as their highly polar C–O bonds induce large dipole moment changes during vibration, whereas these modes are weak in the Raman spectra due to low changes in polarizability. This results in a broad, multipeak band between 1150 and 970 cm^–1^. Carbohydrate −OH vibrations appeared as a broad band around 3300 cm^–1^, and a band at ∼1542 cm^–1^ originated from C–N vibrations. In GSL with five terminal deuterium atoms in a fatty acid chain, band positions remained unchanged, with only minor intensity increases. Similar to SM-d_9_, such a low deuterium content produces only weak C–D bands in the 2300–2000 cm^–1^ range. In the IR spectra, deuteration slightly modifies the −C–O–C band shape without affecting other features, with a faint signal also in the 2200–2000 cm^–1^ range. Comparison of band shapes in the 2200–2000 cm^–1^ region (Figure S5D) shows that SM-d_9_, SM-*d*_31_, and CER-*d*_31_ share similar Raman profiles, whereas GSL-d_9_ displays a distinctly different band arrangement. In the IR spectra, SM-*d*_31_ and CER-*d*_31_ exhibit the same set of bands, while GSL-d_5_ and SM-d_9_ share a similar pattern. These observations indicate that the spectral features were influenced by the degree of deuterium substitution.

For the investigated SM, CER, and GSL, in addition to the characteristic C–D stretching vibrations of their deuterated forms (2220–2050 cm^–1^), several specific marker bands can be identified based on structural differences and vibrational mode activity in Raman and IR spectra. SM exhibits a distinctive Raman band at 718 cm^–1^, attributed to the νs­(N^+^(CH_3_)_3_–C) vibration of the phosphocholine headgroup, and an IR band at 1230 cm^–1^ corresponding to phosphate stretching. In the CER, a sharp and intense 3289 cm^–1^ band in the IR spectrum, assigned to ν­(−NH) stretching, serves as a characteristic indicator. GSLs are most easily recognized in the IR spectra due to a broad band in the 1150–1030 cm^–1^ region, arising from polar ν­(C–O–C) vibrations of glycosidic moieties.

The collected Raman and IR spectra of deuterated sphingolipids emphasize their potential as diagnostic markers of stress-related lipid remodeling and signaling. By including these data in the spectral library, sphingolipid-specific vibrational fingerprints can be used to monitor cellular responses and metabolic alterations in both artificial membranes and living cells, directly supporting the study’s aim to create a comprehensive reference for label-free lipid imaging.

### C–D Intensity Signatures as Semi-Quantitative Semiuantitative Probes of Lipid Remodeling

As discussed above, the most informative spectral region for detecting the presence, quantity, and alterations of selected lipid probes is present between 2300 and 2000 cm^–1^, as it does not overlap with bands from endogenous components of the investigated samples. Nevertheless, in the case of the active probes investigated in these studies, where isotopic substitution exceeds 30 deuterium atoms, pronounced alterations arise not only within the C–D stretching region but also across multiple spectral domains.

Based on the structural formulas of the deuterated lipids (Figure S6), the spectral alterations were rationalized as a function of both the number and the specific positions of the substituted deuterium atoms. Full deuteration of palmitic and stearic acids (Figure 1, PA-*d*_31_ and SA-*d*_35_) resulted in systematic shifts in both Raman and IR spectra, caused a reduction in vibrational frequencies, and modified mode coupling along the acyl chains.^63^ As a consequence, the C–H stretching modes were displaced to the silent 2100–2300 cm^–1^ region, and a measurable redistribution of intensities within the fingerprint domain was also observed (RS: 1130, 989, and 917 cm^–1^; IR: 1092 and 1054 cm^–1^). Comparable effects were detected for phospholipids (Figure 3, PC-*d*_70_ and PC-*d*_83_). Isotopic substitution of both hydrocarbon chains shifted the CH_2_/CH_3_-associated modes toward lower wavenumbers (e.g., Raman 1092 and 984 cm^–1^), while the characteristic vibration of the trimethylammonium group (N^+^(CH_3_)_3_) remained unchanged.^64^ When the headgroup was also deuterated (PC-*d*_83_), this band was likewise shifted (e.g., Raman 640 cm^–1^), confirming its sensitivity to isotopic substitution. These observations are summarized in Table 2.

**Table 2:** Vibrational Assignments of C–H/C–D-Related Bands Follow Established Raman and IR Analyses of Fully Deuterated Lipids^a^

Raman [cm^–1^]									
**lipids**	**v**_**as**_ **CH**_**3**_	**v**_**as**_ **CD**_**3**_	**v**_**as**_ **CH**_**2**_	**v**_**as**_ **CD**_**2**_	**v**_**s**_ **CH**_**3**_	**v**_**s**_ **CD**_**3**_	**v**_**s**_ **CH**_**2**_	**v**_**s**_ **CD**_**2**_	**mixed C–C skeletal modes with dominant CH**_**2**_ **/CD**_2_ **contributions**
PA	2953		2928		2880		2850		1462, 1440, 1420, 1130, 1062, 989, 917
PA-d_31_		2200		2123		2102		2073	1305, 1251, 1149, 1057, 993, 917, 829
SA	2953		2928		2880		2850		1462, 1440, 1420, 1130, 1062, 989, 917
SA-d_35_		2200		2123		2102		2073	1305, 1251, 1149, 1057, 993, 917, 829
PC	2960		2935		2892		2850		1461, 1438, 1296, 1129,1105, 1062, 718 (v_s_N^+^(CH_3_)_3_)
PC-d_70_		2200		2180		2106		2080	1146, 986, 918, 832, 718 (v_s_N^+^(CH_3_)_3_)
PC-d_83_		2200		2180		2106		2080	1146, 986, 918, 832, 639 (v_s_N^+^(CD_3_)_3_)

IR [cm^–1^]									
**lipids**	**v**_**as**_ **CH**_**3**_	**v**_**as**_ **CD**_**3**_	**v**_**as**_ **CH**_**2**_	**v**_**as**_ **CD**_**2**_	**v**_**s**_ **CH**_**3**_	**v**_**s**_ **CD**_**3**_	**v**_**s**_ **CH**_**2**_	**v**_**s**_ **CD**_**2**_	**mixed C–C skeletal modes with dominant CH**_**2**_ **/CD**_2_ **contributions**
PA	2958		2919		2872		2850		1471, 1463, 1404, 1307, 1296
PA-d_31_		2213		2193		2156		2088	1433, 1410, 1332, 1306, 1085,1057
SA	2958		2919		2872		2850		1471, 1463, 1404, 1307, 1296
SA-d_35_		2213		2193		2156		2088	1423, 1411, 1319, 1293, 1091,1058
PC	2956		2924		2878		2850		1487, 1417, 1376, 1169, 970 (v_s_N^+^(CH_3_)_3_), 926
PC-d_70_		2214		2193		2155		2088	1482, 1417, 1331, 970 (v_s_N^+^(CH_3_)_3_), 923
PC-d_83_		2214		2193		2155		2088	1187, 1179, 1148, 1017, 900

a The modes observed in the 1200–800 cm^–1^ region originate mainly from mixed C–C skeletal vibrations with dominant CH_2_/CD_2_ rocking, twisting, and wagging contributions.

In contrast, selective deuteration of only one hydrocarbon chain, as in SM-*d*_31_ and CER-*d*_31_ (Figure 4), led predominantly to an enhancement of intensities within the fingerprint region (RS 1672–1063 cm^–1^; IR 1645–970 cm^–1^) and to the appearance of additional Raman modes below ∼983 cm^–1^ attributed to deformations of the deuterium-substituted aliphatic chain. Variations in both intensity and wavenumber within the C–D stretching region (Figure S5) were further indicative of conformational rearrangements of the lipid backbone, demonstrating that isotopic substitution affected not only the vibrational signatures of individual functional groups but also the collective molecular configuration.^21^

By integrating the area under the C–D bands, semiquantitative data on the incorporated probe can be obtained. Importantly, for lipid species in which both C–D and C–H stretching bands are present, the integrated C–D/C–H intensity ratios were additionally calculated following the classical normalization approach and are presented in the Supporting Information (Figure S7). Integration of the C–D band area provides a reliable semiquantitative readout of probe incorporation and lipid remodeling, offering a powerful analytical tool. This approach enables direct assessment of lipid organization, domain formation, and drug–lipid interactions under varying physiological or experimental conditions. The C–D/C–H ratio not only confirms the formation of newly generated C–D bonds but also serves as an internal calibration reference that prevents overestimation of spectral shifts and enables the quantitative evaluation of the fraction of transformed molecules.^21^ Moreover, an important consideration when using deuterated lipids as metabolic tracers is the kinetic isotope effect (KIE).^26,65^ Substitution of C–H bonds with C–D bonds increases the bond strength and can reduce the rates of biochemical reactions.

Direct comparison of Figures 5 and S7 demonstrates that the trends obtained from C–D/C–H ratios closely mirror those derived from C–D band integration alone, confirming the robustness and internal consistency of the adopted analytical strategy. Notably, analysis based solely on C–D band integrals enables a direct and consistent comparison across both partially and fully deuterated lipid species. In the case of fully deuterated lipids, C–H stretching vibrations are absent, rendering the calculation of a C–D/C–H ratio impossible. Consequently, the use of C–D integrals provides a uniform quantitative metric applicable to all investigated lipid probes, allowing meaningful interlipid comparisons independent of the degree of deuterium substitution.

**Figure 5:**
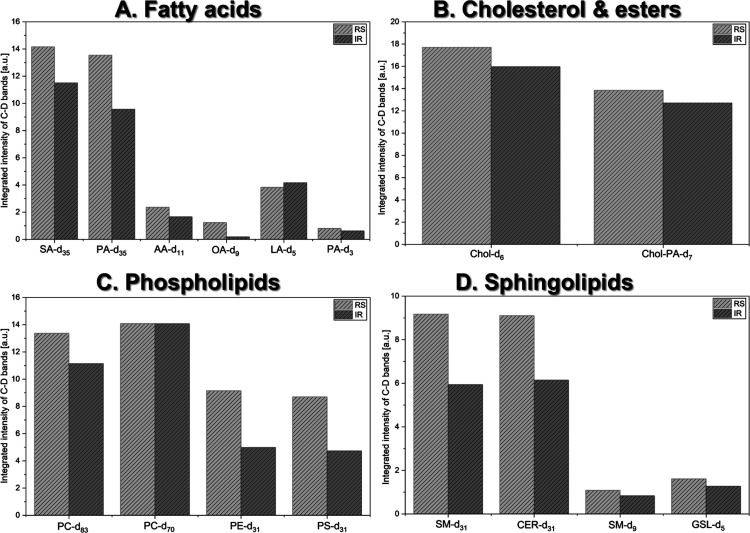
Calculated integral intensities for C–D bands in the 2300–2000 cm^–1^ spectral region for all investigated deuterated compounds: fatty acids (A), cholesterol and esters (B), phospholipids (C), and sphingolipids (D). Intensities of these bands were correlated for both spectroscopic techniques—RS (532 nm) and IR.

As observed, the band intensity correlates directly with the number of C–D groups introduced into the lipid molecules (Figure 5). To semiquantitatively assess this relationship, the linear dependence between the number of deuterated atoms and the integrated Raman (Figure S8­(1A–C)) and IR (Figure S8­(2A–C)) intensities of the C–D bands was determined. The strong correlations (*R* ^2^ values) confirm a proportional signal enhancement with increasing deuterium content. For fatty acids (Figure 5A), PA-d_31_ and SA-d_35_ exhibited comparable intensities in both Raman and IR spectra, whereas lipids containing only 3–11 deuterium atoms displayed substantially lower values. The linear trend demonstrates an increasing signal intensity with higher degrees of deuterium substitution. For this group, the linear fitting yielded *R* ^2^ = 0.9312 for Raman and *R* ^2^ = 0.8569 for IR, reflecting lower IR sensitivity relative to RS. In cholesterol and cholesteryl palmitate (Figure 5B), which possess a similar number of deuterium atoms (Chol-d_6_ and Chol-PA-d_7_), the C–D signal is more pronounced for Chol-d_6_, consistent with the spectral profiles in Figure 2. This difference likely arises because, in Chol-PA, the long-chain palmitic acid moiety may attenuate the C–D signal, whereas in Chol-d_6_, deuteration occurs solely within the single side chain. A similar trend was observed for both the RS and IR techniques. For deuterated phospholipids (Figure 5C), where the 83, 71, and 31 deuterium atoms are presented in the structure, the correlation coefficients were 0.9067 and 0.8022 for RS and IR, respectively. In Figure 5D, with 31, 9, and 5 substituted ^1^H, the relationships present similar values for both RS (0.9729) and IR (0.9675), confirming that the C–D band intensity consistently scales with the degree of ^1^H to ^2^H substitution across all analyzed lipids (Figure S8).

Overall, these results demonstrate that deuterium substitution induces systematic, predictable, and lipid-class-dependent modifications across multiple vibrational regions, extending beyond the C–D stretching window. The strong linear relationship between the C–D band intensity and the degree of deuteration confirms that vibrational spectroscopy provides a robust semiquantitative framework for comparing structurally diverse lipid probes. Together, these findings validate the use of deuterated lipids as reliable spectroscopic reporters of lipid composition, organization, and remodeling, establishing a solid reference basis for future studies of complex biological systems.

## Conclusions

The analysis of C–D vibrational bands in the 2300–2000 cm^–1^ range proved to be a highly sensitive and selective approach for monitoring the incorporation and distribution of deuterated lipid probes across different lipid classes. This spectral window, free from overlapping endogenous vibrations, enabled a semiquantitative determination of the deuterium content through band area integration, revealing a clear correlation between the C–D band intensity and the degree of hydrogen-to-deuterium substitution. Fatty acids with a high level of deuteration (PA-d_31_, SA-d_35_) exhibited markedly stronger C–D signals than their partially substituted analogues, with consistent trends observed in both Raman and IR spectra. Differences between cholesterol and cholesteryl palmitate further demonstrated that the molecular structure and the localization of deuterium atoms influence the C–D vibrational response, reflecting variations in the molecular environment and vibrational coupling. Phospholipids and sphingolipids followed similar dependences, confirming the broad applicability of this approach across diverse lipid types. A comprehensive summary of the Raman and IR band assignments for all investigated lipids and their deuterated analogs is provided in the Supporting Information (Tables S1–S3).

By the introduction of deuterated lipids into cells or model systems such as liposomes and membranes, the resulting C–D signatures enable quantitative tracking of lipid remodeling and the effects of external factors, including drugs, radiation, or oxidative stress, on membrane components. Moreover, these deuterated probes can be visualized and monitored noninvasively by using spectroscopic imaging techniques such as IR and RS, allowing the simultaneous observation of lipid distribution and chemical composition changes within intact biological structures.

Overall, the presented methodology establishes a versatile experimental framework for developing and applying spectroscopically active probes. While this study focuses on establishing the experimental foundation, future work may incorporate theoretical spectral simulations to complement and expand the current data set. The broader potential of this approach extends beyond fundamental biophysical studies to future spectrolipidomics, drug testing, and therapeutic screening, where noninvasive and chemically specific visualization of lipid dynamics will be essential for understanding and modulating cellular responses at the molecular level.

## Supplementary Material



## Notes

Additional data supporting the main findings of the study: Raman spectra of selected fatty acids, cholesterol and selected cholesteryl ester, phospholipids, and sphingolipids and their deuterated forms measured with 633 and 785 nm laser lines (Figures S1–S4); 2300–2000 cm^–1^ spectral range for the Raman (532 nm) and IR spectra of deuterated lipids (Figure S5); structural formulas of selected deuterated lipids (Figure S6); calculated integral intensities for C–D/C–H band ratios in the 2300–2000 and 3000–2800 cm^–1^ spectral regions for selected deuterated compounds for RS (532 nm) and IR (Figure S7); linear dependence between the number of deuterated atoms and the integrated Raman and IR intensities of the C–D bands with correlation (*R* ^2^) (Figure S8); summary of band assignment for lipids and their deuterated forms for Raman and IR in the 3500–2050, 1800–1100, and 1100–700 cm^–1^ spectral ranges (Tables S1–S3) (PDF)
