# Supplementary material for: Spectroscopic Active Probes for Investigation of Lipid Transformation in Cells and Membranes
Source: J Phys Chem B. 2026 Jan 27;130(6):1743–54. doi: 10.1021/acs.jpcb.5c05981 (PMC13296733; doi:10.1021/acs.jpcb.5c05981)
Supplement: Supplementary file 1 [file jp5c05981_si_001.pdf]

Supporting Information

Spectroscopic Active Probes for Investigation of Lipids Transformation in Cells and Membranes

K. Chrabąszcz*

Institute of Nuclear Physics, Polish Academy of Sciences
Radzikowskiego 152, 31-342 Krakow, Poland
*karolina.chrabaszcz@ifj.edu.pl

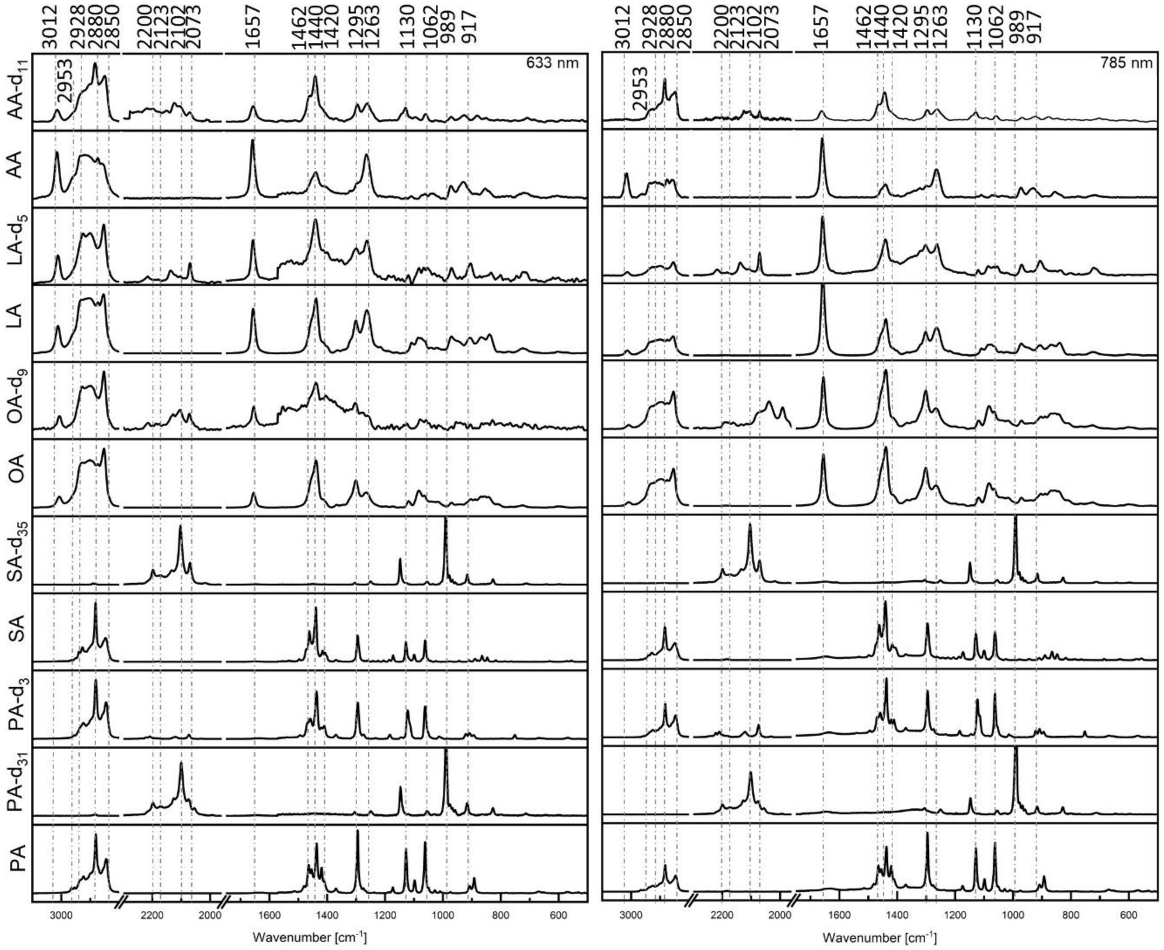

Figure S1. Raman spectra of selected fatty acids and their deuterated forms measured with 633 nm and 785nm laser line.

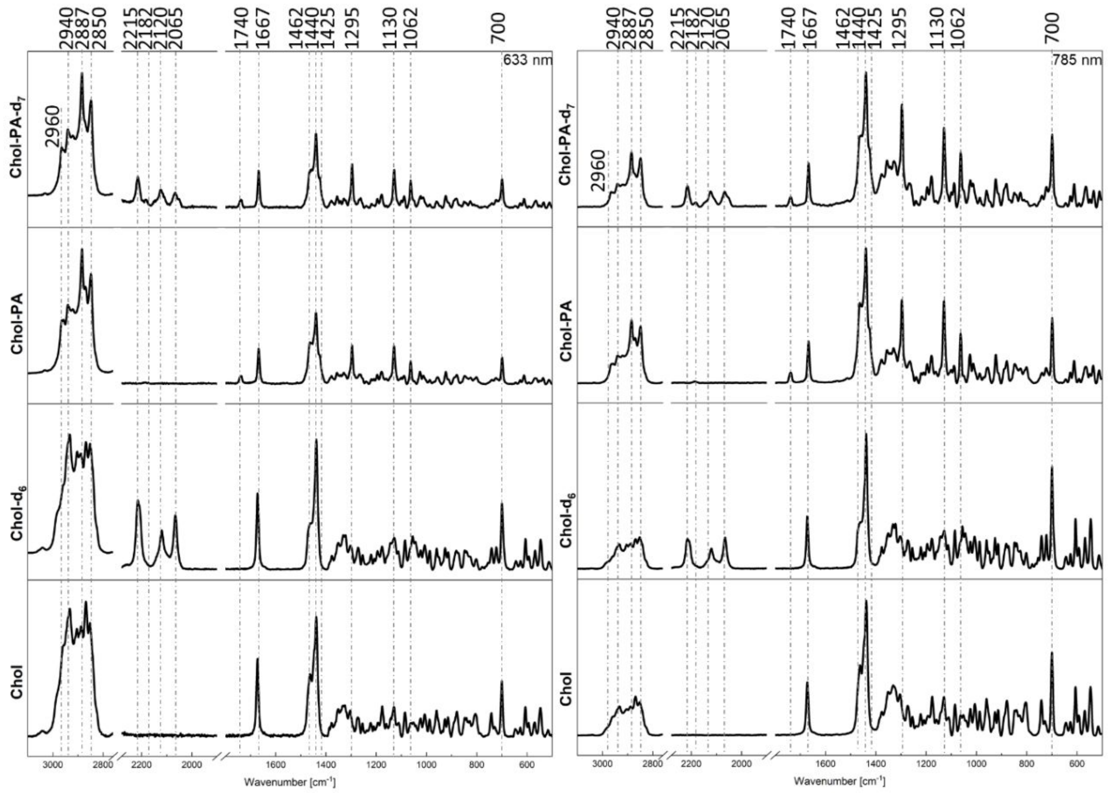

Figure S2. Raman spectra of cholesterol and selected cholesteryl ester with their deuterated forms measured with 633 nm and 785nm laser line.

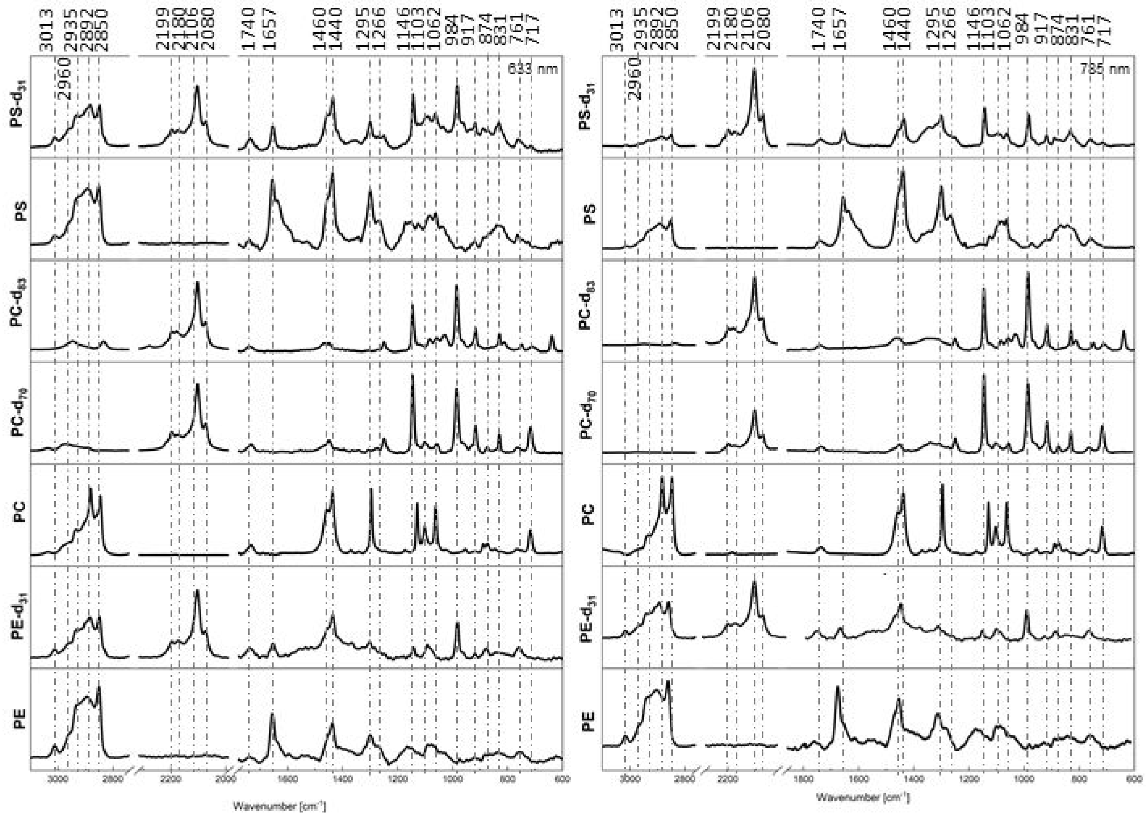

Figure S3. Raman spectra of selected phospholipids with their deuterated forms measured with 633 nm and 785nm laser line.

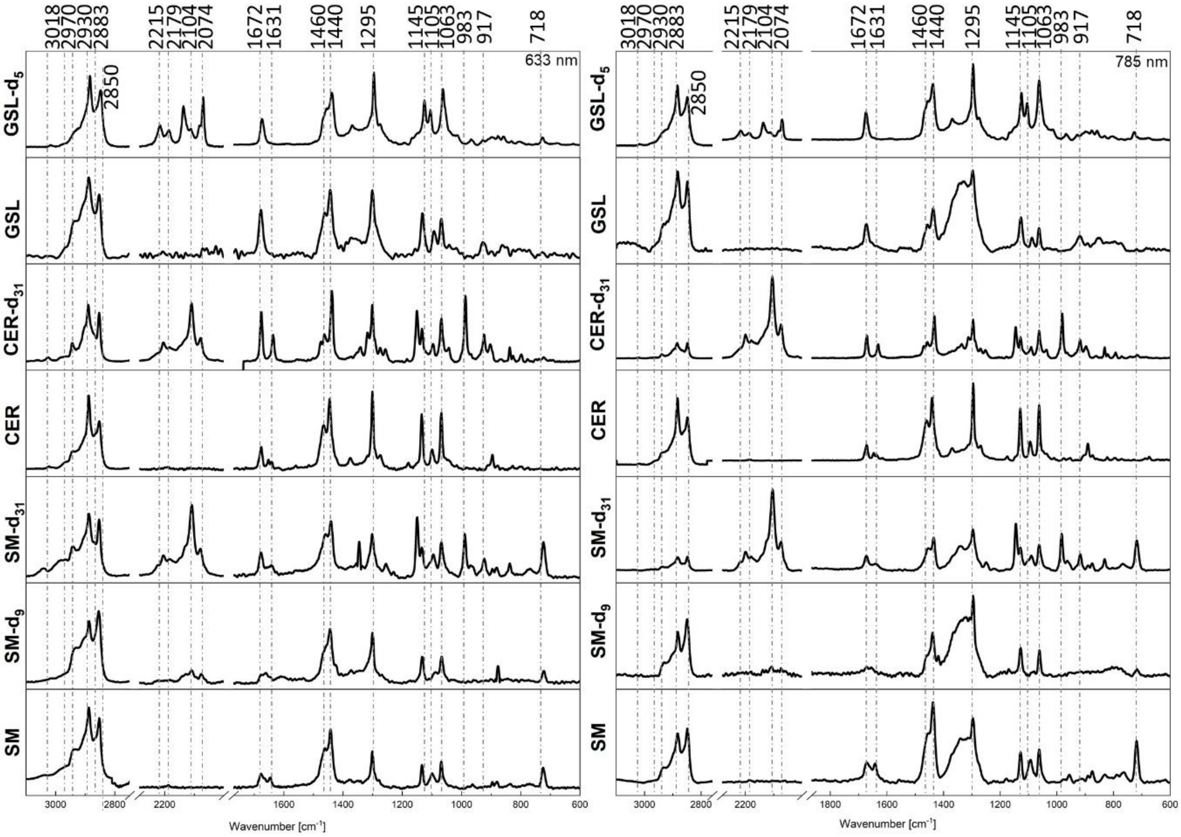

Figure S4. Raman spectra of selected sphingolipids with their deuterated forms measured with 633 nm and 785nm laser line.

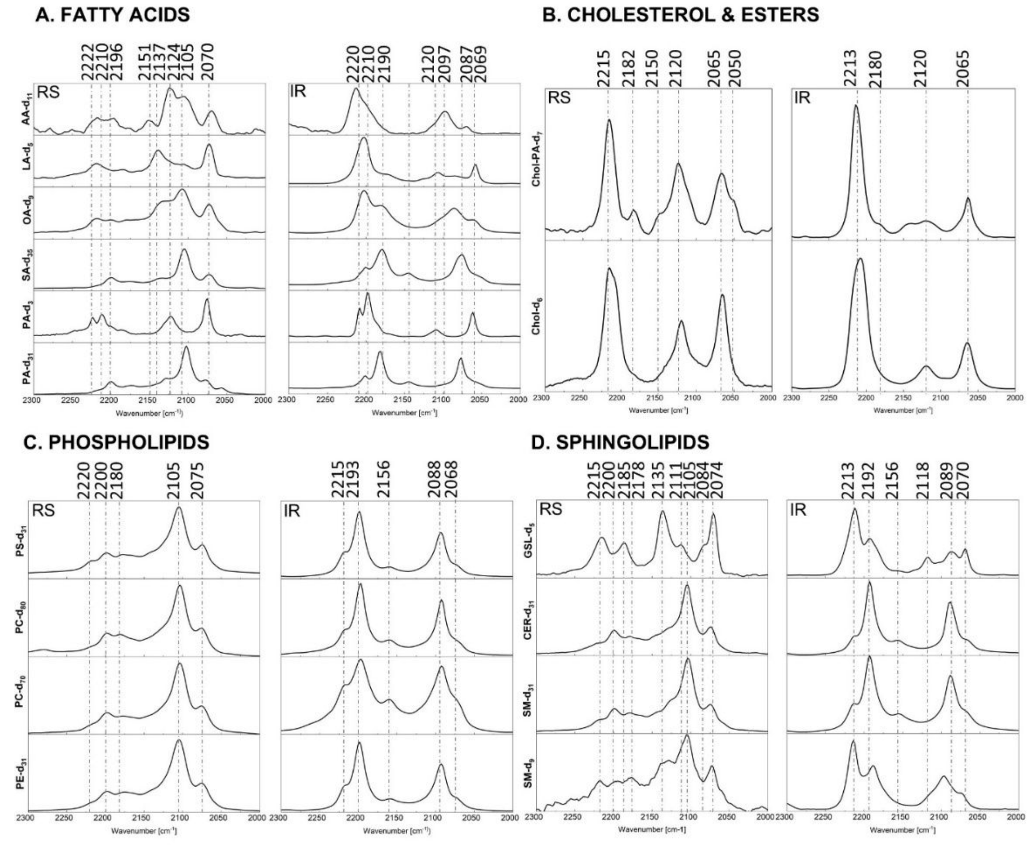

Figure S5. The 2300-2000 cm^{-1} spectral range for Raman (532nm) and IR spectra of deuterated lipids.

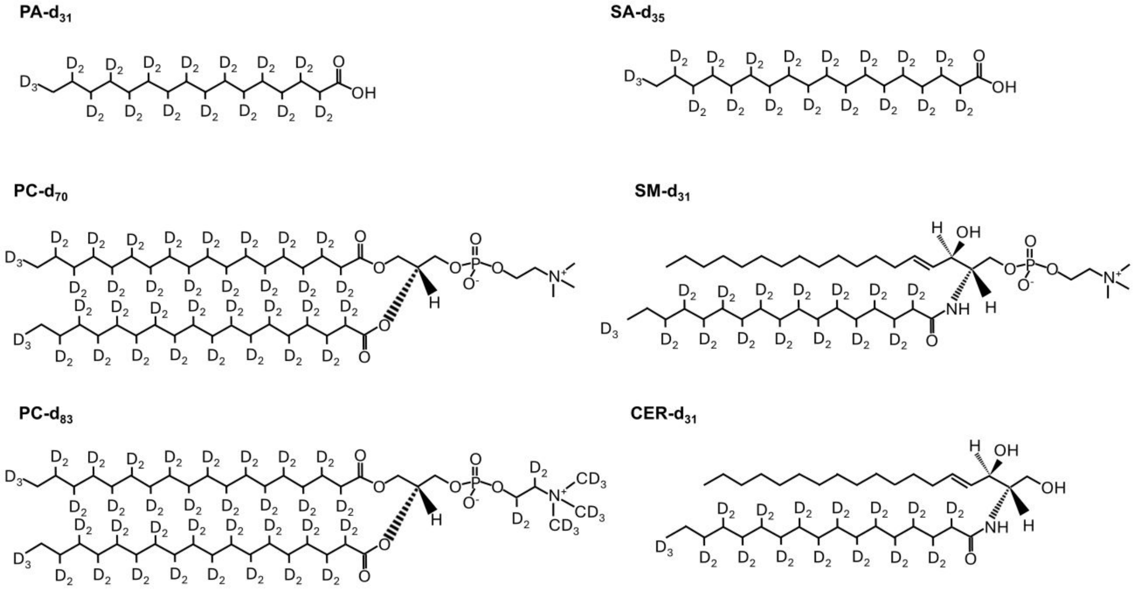

Figure S6. The structural formulas of selected deuterated lipids.

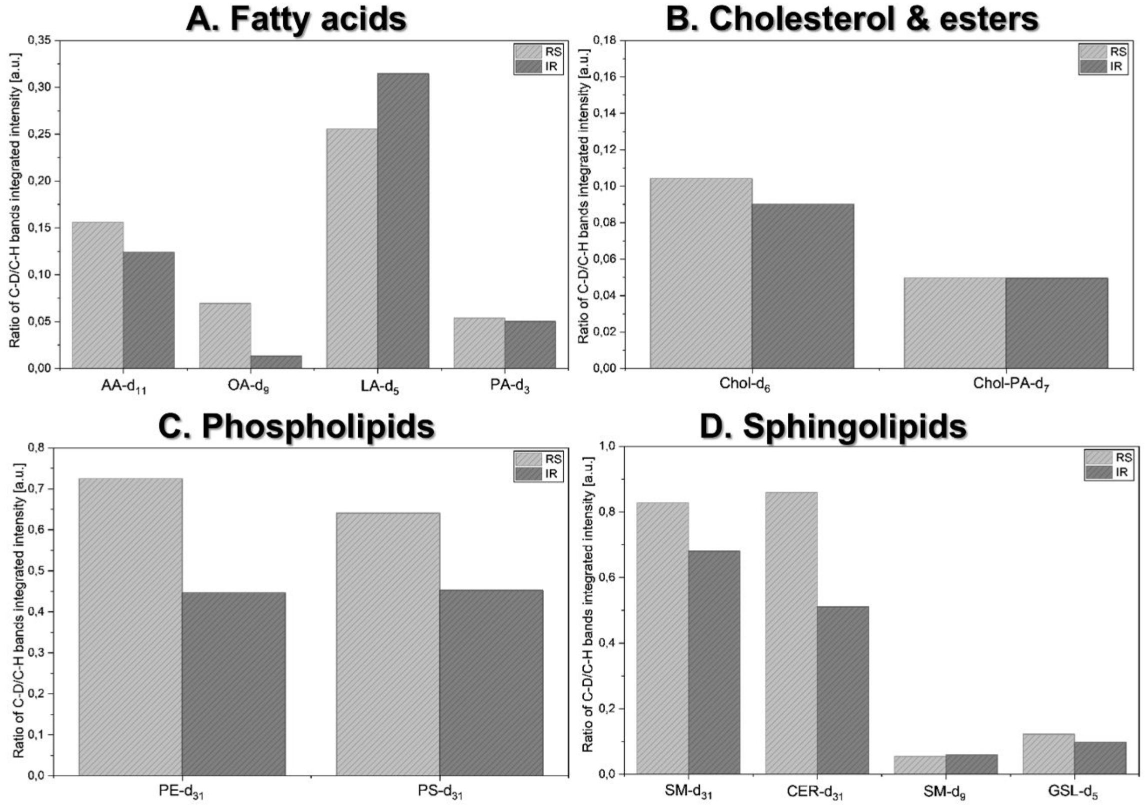

Figure S7. Calculated integral intensities for C–D/C–H bands ratio in the 2300-2000 cm⁻¹ and 3000-2800 cm⁻¹ spectral region for selected deuterated compounds: fatty acids (A), cholesterol and esters (B), phospholipids (C) and sphingolipids (D). Intensities of these bands were correlated for both spectroscopic techniques – RS (532 nm) and IR.

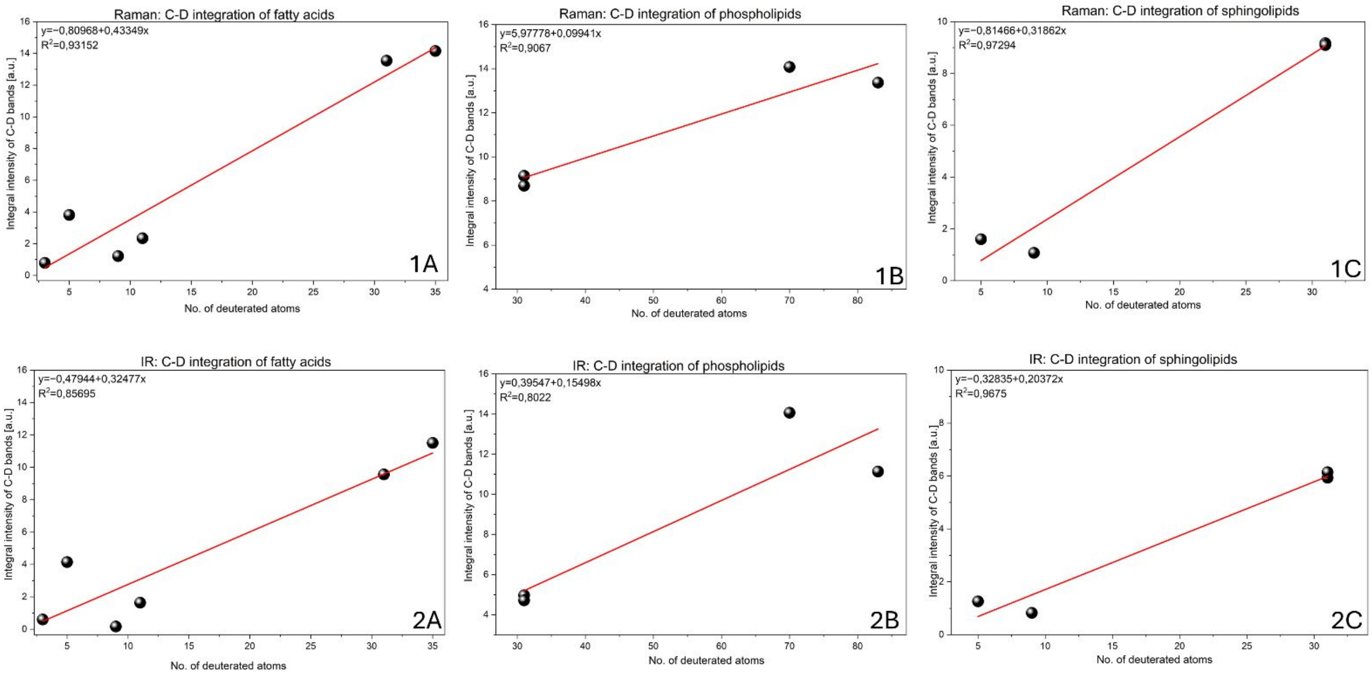

Figure S8. Linear dependence between the number of deuterated atoms and the integrated Raman (1A-C) and IR (2A-C) intensity of the C–D bands. The strong correlation (R^2) confirms proportional signal enhancement with increasing deuterium content.

Table S1. The summary of bands assignment for lipids and their deuterated forms for Raman and IR in the 3500 cm^{-1} - 2050 cm^{-1} spectral range. Band positions for IR are listed in brackets.

	Summary of Vibrational Band Assignments for Lipids and Their Deuterated Analogs (3500 cm^{-1} - 2050 cm^{-1})												
		v(OH)	v(3βOH)	v(NH)	=(CH)	$v_{as}(CH_3)$	$v_{as}(CH_2)$	$v_s(CH_3)$	$v_s(CH_2)$	$v_{as}(CD_3)$	$v_{as}(CD_2)$	$v_s(CD_3)$	$v_s(CD_2)$
**Fatty acids**	**PA**					2953 (2958)	2928 (2872)	2880 (2919)	2850				
	**PAd₃₁**									2200 (2213)	2123 (2193)	2102 (2156)	2073 (2088)
	**PAd₃**					2953 (2958)	2928 (2872)	2880 (2919)	2850	2200 (2213)	2123 (2193)	2102 (2156)	2073 (2088)
	**SA**					2953 (2958)	2928 (2872)	2880 (2919)	2850				
	**SAd₃₅**									2200 (2213)	2123 (2193)	2102 (2156)	2073 (2088)
	**OA**				3012	2953 (2958)	2928 (2872)	2880 (2919)	2850				
	**OAd₉**									2200 (2213)	2123 (2193)	2102 (2156)	2073 (2088)
	**LA**												
	**LAd₅**									2200 (2213)	2123 (2193)	2102 (2156)	2073 (2088)
	**AA**												
	**AAd₁₁**									2200 (2213)	2123 (2193)	2102 (2156)	2073 (2088)
**Cholesterol & esters**	**Chol**	(3444)	(3360)				2940 (2920)		2850 (2850)				
	**Chold₆**									2215 (2215)		2120 (2120)	2065 (2065)
	**Chol – PA**					2960 (2940)		2887 (2870)					
	**Chol– PAd₇**									2215 (2215)	2182 (2180)	2120 (2120)	2065 (2065)
**Phospholipids**	**PE**				3013 (3013)	2960 (2956)	2935 (2924)	2892 (2878)	2850 (2850)				
	**PEd₃₁**									2200 (2214)	2180 (2193)	2106 (2155)	2080 (2088)
	**PC**	(3382)				2960 (2956)	2935 (2924)						
	**PCd₇₀**									2200 (2214)	2180 (2193)	2106 (2155)	2080 (2088)
	**PCd₈₃**									2200 (2214)	2180 (2193)	2106 (2155)	2080 (2088)
	**PS**				3013 (3013)	2960 (2956)	2935 (2924)	2892 (2878)	2850 (2850)				
	**PSd₃₁**									2200 (2214)	2180 (2193)	2106 (2155)	2080 (2088)
**Sphingolipids**	**SM**	(3355)		(3289)		2970 (2954)	2930 (2920)	2883 (2873)	2850 (2850)				
	**SMd₉**									2215 (2213)	2179 (2192)	2104 (2089)	2074 (2067)
	**SMd₃₁**												
	**CER**												
	**CERd₃₁**									2215 (2213)	2179 (2192)	2104 (2089)	2074 (2067)
	**GSL**												
	**GSLd₅**									2215 (2213)	2179 (2192)	2104 (2089)	2074 (2067)

Table S2. The summary of bands assignment for lipids and their deuterated forms for Raman and IR in the 1800 cm^{-1} - 1100 cm^{-1} spectral range. Band positions for IR are listed in brackets.

Summary of Vibrational Band Assignments for Lipids and Their Deuterated Analogs (1800 cm^{-1} - 1100 cm^{-1})

		(C=O)	(C=O), δ(N-H)	ν(C=C)	δ(N-H), ν(C-N)	(CH_2/ CH_3)			δ(=CH), ρ(CH)	$\nu_{as}(PO_2^-)$	ν(C-C)
Fatty acids	PA					1462 (1463)	1440 (1404)	1420 (1307)	1295		1130,1060
	PAd31										1130
	PAd3					1462 (1463)	1440 (1404)	1420 (1307)	1295		1130,1060
	SA					1462 (1463)	1440 (1404)	1420 (1307)	1295		1130,1060
	SAd35	1710									1130
	OA										
	OAd9								1295, 1263		
	LA						1440 (1404)	1420 (1307)			
	LAd5			1657							
	AA								1263		
	AAd11					1462 (1463)			1295, 1263		1130
Cholesterol & esters	Chol					1462 (1463)	1440 (1434)				1130,1060
	Chold6										
	Chol – PA			1667 (1671)							
	Chol– PAd7	1740 (1740)				1462 (1463)	1440 (1434)	1425 (1376)			
Phospholipids	PE			1657 (1650)					1295, 1266		
	PEd31					1460 (1470)	1440 (1420)				1146
	PC								1295		
	PCd70	1740 (1735)								1250	1146
	PCd83										
	PS			1657 (1650)		1460 (1470)	1440 (1420)		1295, 1266		
	PSd31										1146
Sphingolipids	SM									1230	
	SMd9										
	SMd31				(1542)						
	CER		1672 (1645)			1460 (1464)	1440 (1431)		1295		1145,1063
	CERd31										
	GSL										
	GSLd5										

Table S3. The summary of bands assignment for lipids and their deuterated forms for Raman and IR in the 1100 cm^{-1} - 700 cm^{-1} spectral range. Band positions for IR are listed in brackets.

<table>
<tr><th colspan="12">Summary of Vibrational Band Assignments for Lipids and Their Deuterated Analogs (1100 cm^{-1} – 700 cm^{-1})</th></tr>
<tr><th></th><th></th><th>ν_{as}(C-O)</th><th>ν(P-O)</th><th>ν_s(C-O)</th><th>β(CH)</th><th>$\nu_{as}N^+(CH_3)_3$</th><th>β(-C=C-)</th><th>δ(C-H)</th><th>Chol ring deformation</th><th>$\nu_sN^+(CH_3)_3$</th><th>$\nu N^+(CH_3)_3$</th></tr>
<tr><td rowspan="11">Fatty acids</td><td>PA</td><td></td><td></td><td></td><td>989, 917</td><td></td><td rowspan="11">(942)</td><td></td><td></td><td></td><td></td></tr>
<tr><td>PAd_{31}</td><td></td><td></td><td>(1092)</td><td></td><td></td><td></td><td></td><td></td><td></td></tr>
<tr><td>PAd_3</td><td></td><td></td><td></td><td></td><td></td><td></td><td></td><td></td><td></td></tr>
<tr><td>SA</td><td>(1121)</td><td></td><td></td><td></td><td></td><td></td><td></td><td></td><td></td></tr>
<tr><td>SAd_{35}</td><td></td><td></td><td>(1092)</td><td></td><td></td><td></td><td></td><td></td><td></td></tr>
<tr><td>OA</td><td></td><td></td><td></td><td></td><td></td><td></td><td></td><td></td><td></td></tr>
<tr><td>OAd_9</td><td></td><td></td><td></td><td></td><td></td><td></td><td></td><td></td><td></td></tr>
<tr><td>LA</td><td></td><td></td><td></td><td></td><td></td><td></td><td></td><td></td><td></td></tr>
<tr><td>LAd_5</td><td></td><td></td><td></td><td></td><td></td><td></td><td></td><td></td><td></td></tr>
<tr><td>AA</td><td></td><td></td><td></td><td></td><td></td><td></td><td></td><td></td><td></td></tr>
<tr><td>AAd_{11}</td><td></td><td></td><td></td><td></td><td></td><td></td><td></td><td></td><td></td></tr>
<tr><td rowspan="4">Cholesterol & esters</td><td>Chol</td><td></td><td></td><td rowspan="2">(1055)</td><td rowspan="4">989, 917</td><td></td><td rowspan="2">955</td><td rowspan="2">837</td><td rowspan="4">700</td><td></td><td></td></tr>
<tr><td>$Chold_6$</td><td></td><td></td><td></td><td></td><td></td></tr>
<tr><td>Chol – PA</td><td rowspan="2">(1164)</td><td></td><td></td><td></td><td></td><td></td><td></td><td></td></tr>
<tr><td>Chol– PAd_7</td><td></td><td></td><td></td><td></td><td></td><td></td><td></td></tr>
<tr><td rowspan="7">Phospholipids</td><td>PE</td><td rowspan="7">(1170)</td><td rowspan="7">1092 (1082)</td><td rowspan="7">(1040)</td><td></td><td rowspan="7">874</td><td></td><td></td><td></td><td></td><td></td></tr>
<tr><td>PEd_{31}</td><td>984, 917</td><td></td><td></td><td></td><td></td><td></td></tr>
<tr><td>PC</td><td></td><td></td><td></td><td></td><td></td><td rowspan="2">717, (970)</td></tr>
<tr><td>PCd_{70}</td><td rowspan="2">984, 917</td><td></td><td></td><td></td><td></td></tr>
<tr><td>PCd_{83}</td><td></td><td></td><td></td><td></td><td>640</td></tr>
<tr><td>PS</td><td></td><td></td><td></td><td></td><td></td><td></td></tr>
<tr><td>PSd_{31}</td><td>984, 917</td><td></td><td></td><td></td><td>761,717</td><td></td></tr>
<tr><td rowspan="7">Sphingolipids</td><td>SM</td><td></td><td rowspan="3">1092 (1080)</td><td></td><td></td><td></td><td></td><td></td><td></td><td rowspan="3">718</td><td rowspan="3">970</td></tr>
<tr><td>SMd_9</td><td></td><td></td><td></td><td></td><td></td><td></td><td></td></tr>
<tr><td>SMd_{31}</td><td></td><td></td><td>984, 917</td><td>880</td><td></td><td></td><td></td></tr>
<tr><td>CER</td><td rowspan="4">(1150)</td><td rowspan="4">1092</td><td></td><td></td><td></td><td></td><td></td><td></td><td></td><td></td></tr>
<tr><td>$CERd_{31}$</td><td></td><td>984, 917</td><td>880</td><td></td><td></td><td></td><td></td><td></td></tr>
<tr><td>GSL</td><td rowspan="2">1030</td><td></td><td></td><td></td><td></td><td></td><td></td><td></td></tr>
<tr><td>$GSLd_5$</td><td></td><td></td><td></td><td></td><td></td><td></td><td></td></tr>
</table>
